# Comparison of Risk and Severity of Helicobacter Pylori Infection in Non-Native Versus US Native Pediatric Patients

**DOI:** 10.1097/PG9.0000000000000331

**Published:** 2023-06-28

**Authors:** Yan Luo, Yinan Fu, Steven Schwarz, Thomas Wallach

**Affiliations:** From the * Department of Pediatrics, SUNY Downstate Health Sciences University; † Department of Pediatric Gastroenterology, Children’s Hospital of Los Angeles; ‡ Division of Pediatric Gastroenterology, Department of Pediatrics, SUNY Downstate Health Sciences University.

**Keywords:** *Helicobacter pylori*, peptic ulcer disease, immigrant, gastritis

## Abstract

**Introduction:**

*Helicobacter pylori* (HP) infection is associated with gastritis, peptic ulcer disease (PUD) in the stomach and duodenum, and an increased risk of gastric cancer. The risk of infection, secondary symptoms, and negative outcomes is known to be increased in low- and middle-income countries and vastly less substantial in the United States and Europe. Current North American Society for Pediatric Gastroenterology, Hepatology, and Nutrition guidelines recommend endoscopic diagnosis and susceptibility-guided therapy, which is not reflected by current adult guidelines for therapy. In this study, we complete a single-center retrospective review of HP risk by nativity status, as well as the results of the use of standard empiric therapy in HP and PUD patients.

**Methods:**

We retrospectively reviewed all endoscopies with patients aged 1–21 years with a known nativity status and identified all HP diagnoses. We also completed the classification of Kyoto scores and classified patients as gastritis versus PUD. Treatment records were obtained, as well as downstream documentation of the impact of empiric therapy. HP prevalence and severity were compared between non-native and native US populations.

**Results:**

In total 332 patients were identified, with 59 HP diagnoses. However, 64 patients were immigrants, and 268 were US natives. Totally 39.1% of all immigrant patients had an endoscopically identified HP infection, compared to only 12.7% of US native patients (*P* < 0.01, relative risk 3.07). HP severity was worse in immigrant patients (Kyoto score 1.5 versus 0.89; *P* = 0.008). Empiric high-dose amoxicillin triple therapy was equally effective in reducing symptoms in gastritis versus PUD patients.

**Conclusions:**

Immigrant patients have a substantially higher risk and severity of HP infection than US natives. Empiric therapy remains highly effective at relieving symptoms. These findings in aggregate suggest that North American Society for Pediatric Gastroenterology, Hepatology, and Nutrition guidelines may not adequately serve non-native pediatric patients, with an additional prospective multicenter study needed to confirm. In addition, a prospective study of treatment based on stool antigen tests, as well as a larger prospective study of empiric therapy, may suggest alterations to our approach in line with recent changes to adult Gastroenterology practice.

**What Is Known**

- Adult gastroenterology societies have revised their guidelines to reflect data that *Helicobacter pylori* (HP) should typically be treated and that susceptibility-guided antibiotic selection is not valuable in the initial treatment.
- HP represents a larger problem in low- and middle-income nations than the United States, and adult guidelines reflect this in suggesting an increased need for suspicion in non-native patients.

**What Is New**

- Non-native pediatric patients in the United States have an extremely high risk of HP and demonstrate increased severity on the Kyoto score.
- Pediatric patients do not demonstrate different outcomes for treatment of typical HP infection and Peptic Ulcer Disease with standard high-dose amoxicillin triple therapy.

## INTRODUCTION

*Helicobacter pylori* (HP) infection is associated both with peptic ulcer disease (PUD) and an increased risk of gastric cancer (1). The severity of infection is heavily linked to associated pathogenicity islands (2), with isolates from the Global South demonstrating higher pathogenicity and US and European isolates with lower pathogenicity (3). At this time, there is a significant amount of debate regarding best practices for HP diagnosis and treatment. The 2016 European Society for Pediatric Gastroenterology, Hepatology, and Nutrition/North American Society for Pediatric Gastroenterology, Hepatology, and Nutrition guidelines recommend that treatment be contingent on the presence of PUD and that endoscopic diagnosis with biopsies and culture for sensitivity should be considered the standard for diagnosis and guiding therapy (4). However, this recommendation differs from other US society recommendations targeted at adults. The Houston guidelines, for example, state that all HP infections be treated and that positive stool antigen testing be considered adequate for empiric therapy (5). The Houston and American College of Gastroenterology guidelines agree on treating all positive patients and also make explicit mention of testing non-native patients, given the higher incidence of high pathogenicity HP in other regions (5,6). Pediatric work has also noted the need to adjust our guidelines by region (7). What data is available regarding current state practice suggests that current adherence to these guidelines is low, with only rare culture-guided therapy and high rates of treatment based on stool antigen testing and breath testing (8,9). However, at this point data studying the specific outcomes of HP infection in immigrant and low-income US pediatric populations is sparse, warranting further evaluation to help inform practice.

The University Hospital at Downstate (UHD) serves a low-income, predominately African-descent community consisting of many non-native families. We clinically experience a high volume of HP disease burden, including abdominal pain and bleeding in pediatric patients, and high rates of gastric cancer in adults. As socioeconomic factors and ethnicity can affect the risk of HP infection, UHD is in a unique position to isolate the impact of nativity on the risk and severity of pediatric HP infection with minimized confounding from socioeconomic status and race/ethnicity. In this study, we retrospectively evaluated our experience with endoscopic HP diagnosis and therapy, assessing the impact of nativity status on the risk of HP infection, and evaluating the outcome of empiric therapy in our population.

## METHODS

This retrospective study was approved by the Institutional Review Board at UHD. We included patients between 1 and 21 years of age who underwent esophagogastroduodenoscopy (EGD) between Jan 2017 and Mar 2022. Patients who had undergone EGD without any gastrointestinal symptoms were excluded, as well as patients where nativity was not documented.

We collected patient demographic characteristics, presenting symptoms, endoscopy reports and histology results, treatment courses, and clinical outcomes. Demographic parameters include age, sex, race, non-native status, and country of origin. Non-native status was defined as being born outside the United States. All patients born in the United States were termed natives. Endoscopy findings such as erythema, nodularity, atrophy, and intestinal metaplasia documented in the report were collected. We assessed the endoscopic activity by using the Kyoto score (10). PUD was defined by the presence of HP infection and ulcerations/erosions, and HP infection was defined by the presence of HP organisms in gastric tissue on immunohistochemical staining. Patient clinical outcomes were reviewed and categorized as “no response”, “improved”, and “resolved” based on patient symptoms after eradication therapy.

HP prevalence was compared in native and non-native groups, and disease severity was assessed using the mean Kyoto score of HP+ patients who were not known or found to have another gastrointestinal condition. Therapeutic efficacy among native and non-native cohorts was determined in all patients not known to have another gastrointestinal condition.

### Statistical Analysis

IBM SPSS Statistics (version 23.0, Chicago, IL) was employed for data analysis. Continuous data were summarized as mean ± standard error and were analyzed by Student’s t-test or Mann–Whitney test when appropriate. Categorical data were presented as frequency (percentage) and evaluated by the Pearson chi-square test or Fisher exact test when appropriate. A *P* value <0.05 was considered statistically significant.

## RESULTS

A total of 332 patients who underwent EGD for Gastroenterology (GI) symptoms during the relevant period met our inclusion criteria. 59 patients (17.8%) were diagnosed with HP infection, and 7 of these were found to have other gastrointestinal diseases, including IBD and Eosinophilic Esophagitis, and were excluded from further analysis. Among a total of 52 HP-positive patients without other GI diseases, 8 had received empiric therapy before endoscopic diagnosis and were also excluded. The demographic characteristics of all patients included in this analysis are summarized in **Table 1**.

**Table 1.** Demographics of patients and clinical presentation

		Non-native (N = 64)	Native (N = 268)	*P*
Age^*^		12.48 ± 3.89	10.56 ± 4.75	0.005
Sex^†^	Female	40 (62.5)	129 (48.1)	
	Male	24 (37.5)	139 (51.9)	0.039
Race^‡^	Black	39 (60.9)	160 (59.7)	
	Caucasian	2 (3.1)	54 (20.1)	
	Asian	10 (15.6)	11 (4.1)	
	Hispanic	7 (10.9)	22 (8.2)	
	Other	6 (9.3)	21 (7.8)	
*H. pylori* infection^†^	Positive	25 (39.1)	34 (12.7)	
	Negative	39 (60.9)	234 (87.3)	<0.01
Country^‡^	Asian country	14 (21.9)	0	
	Caribbean/South/Central American country	33 (51.6)	0	
	African country	6 (9.4)	0	
	US	0	268	
	Europe	1 (1.5)	0	
	Unknown	10 (15.6)	0	
Symptoms^†^	Abdominal pain^†^	49 (76.6)	174 (66.9)	0.136
	Nausea/vomiting^†^	36 (56.3)	129 (49.6)	0.342
	Burning^†^	14 (21.9)	34 (13.0)	0.074
	Belching^†^	3 (4.8)	12 (4.6)	0.98

*H. pylori = Helicobacter pylori.*

^*^Mean ±SD, compared by Mann–Whitney test.

^†^N (%), compared by chi-square test or Fisher exact test if any sample <5.

^‡^N (%).

### Risk by Nativity

**FIGURE 1.**
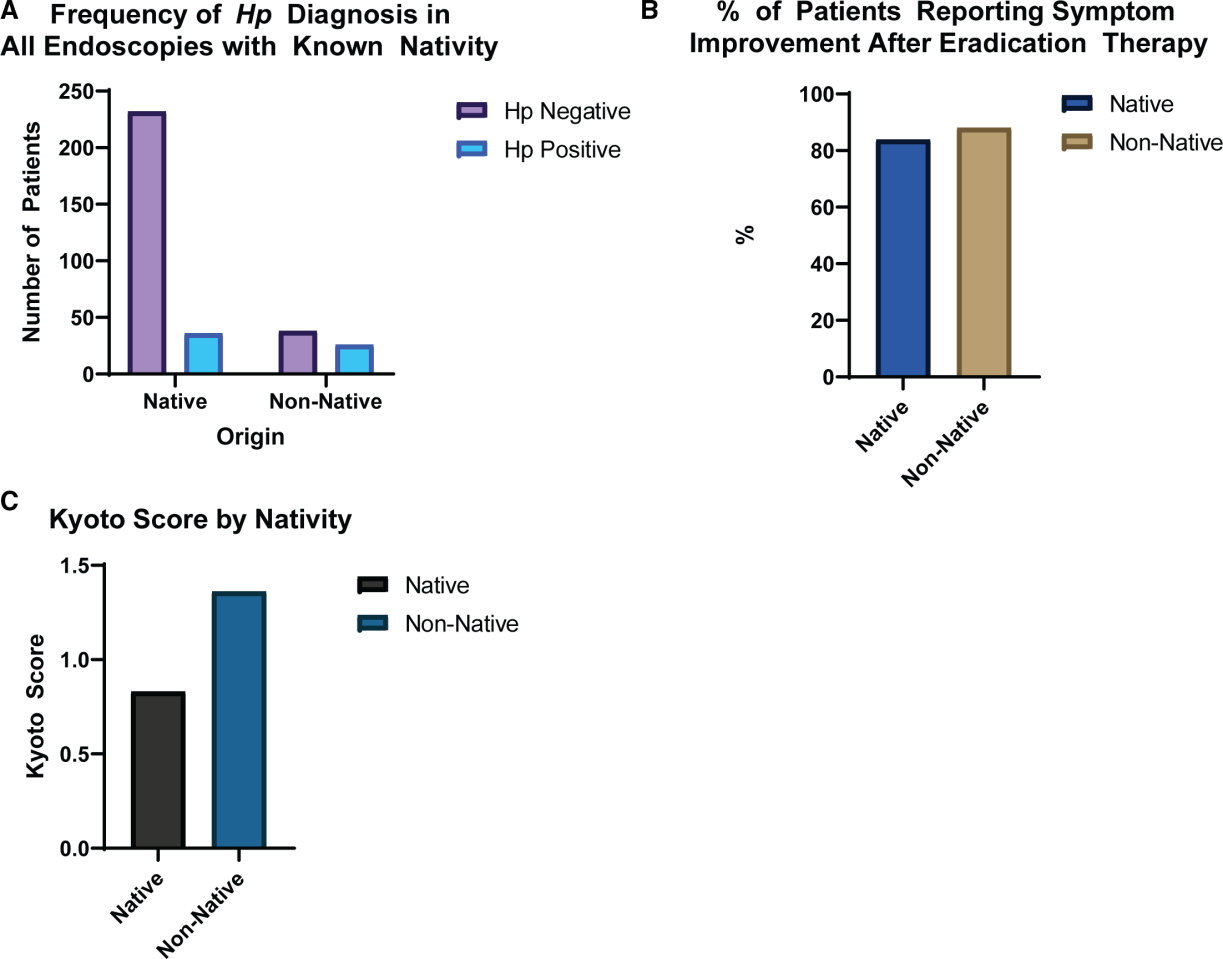
A) Frequency of *Helicobacter pylori* diagnosis in all endoscopies with known nativity. B) Percentage of patients reporting symptom improvement after eradication therapy. C) Kyoto score by nativity.

In our total study population undergoing EGD, 64 patients were non-natives, and 268 patients were US natives. Endoscopic HP diagnosis was markedly different between groups, with an infection rate of 39.1% in non-native versus 12.7% in US native children (*P* < 0.01; relative risk = 3.07) (Fig. 1). In our non-native patients, there was no statistically significant variation noted in the risk of HP infection by region of origin (*P* = 0.65).

### Endoscopic Findings and Therapeutic Outcomes

**FIGURE 2.**
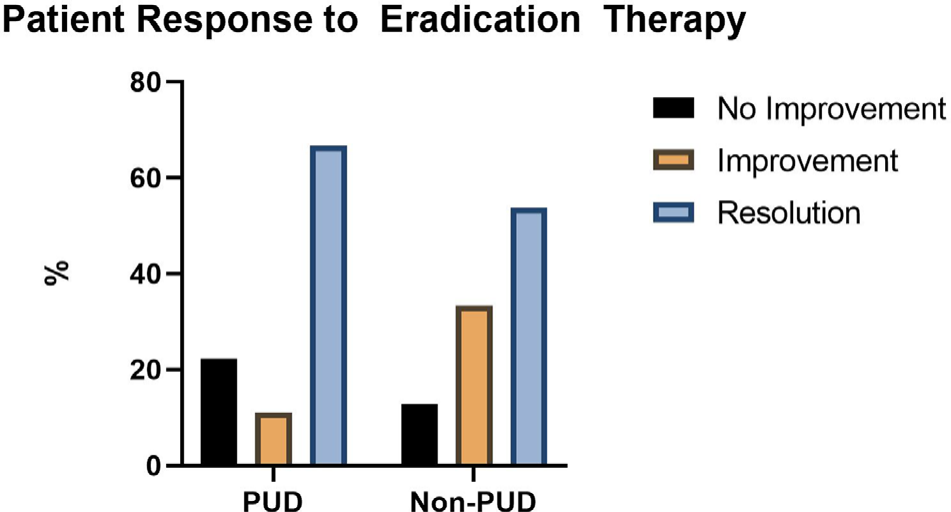
Patient response to *Helicobacter pylori* eradication therapy.

Of the 52 patients with a sole diagnosis of HP infection, no significant differences by nativity were noted in nodularity, but erythema (50% versus 14.3%; *P* = 0.017) and ulceration (34.8% versus 10.3%; *P* = 0.044) were significantly higher in non-native children compared with natives (Fig. 2). Non-native children were also found to have had higher endoscopic disease activity than native children (mean Kyoto score, 1.50 versus 0.89; *P* = 0.008) (Fig. 2). All patients received standard HP eradication therapy of omeprazole, high-dose amoxicillin, and clarithromycin. Of these 44 patients, 41 (85.5%) reported improved or resolved symptoms after completing therapy. Comparison by nativity noted no significant difference by nativity in symptom improvement (Fig. 2). EGD findings are summarized in **Table 2**.

**Table 2.** EGD findings and treatment of *H. Pylori* patients

		Non-native (N = 23)	Native (N = 29)	*P*
Kyoto score^*^		1.50 ± 0.94	0.89 ± 0.57	0.008
Nodularity^†^		16 (80.0)	21 (75.0)	0.68
Erythema^†^		10 (50.0)	4 (14.3)	0.017
PUD^†^		8 (34.8)	3 (10.3)	0.044
Treatment^‡^	Regimen 1	20 (87.0)	27 (93.1)	
	Regimen 2	2 (8.7)	1 (3.4)	
	Regimen 3	0	1 (3.4)	
	Regimen 4	1 (4.3)	0	

EGD = esophagogastroduodenoscopy; *H. pylori = Helicobacter pylori;* PUD = peptic ulcer disease.

^*^Mean ± SD, compared by t-test.

^†^N (%), compared by Fisher exact test.

^‡^N (%).

Regimen1: omeprazole, amoxicillin, and clarithromycin.

Regimen 2: levofloxacin, amoxicillin, and omeprazole.

Regimen 3: omeprazole, metronidazole, and amoxicillin.

Regimen 4: omeprazole, metronidazole, amoxicillin, and bismuth.

We next compared outcomes of therapy based on endoscopic severity. There was no significant difference in the outcome of empiric therapy between PUD and non-PUD patients (*P* = 0.808) (Fig. 2). Further analysis by nativity did not demonstrate any difference between native and non-native PUD patients in symptom improvement with empiric therapy. Therapeutic outcomes are summarized in **Table 3**.

**Table 3.** Clinical outcomes of eradication therapy

	Clinical resolution	Total	Non-PUD	PUD	*P*
All	No response	7 (15.9)	5 (13.9)	2 (25.0)	
	Improved	14 (31.8%)	13 (36.1)	1 (12.5)	
	Resolved	23 (52.3%)	18 (50.0)	5 (62.5)	0.808
Non-native	No response	2 (10.5%)	1 (7.1)	1 (20)	
	Improved	5 (26.3%)	5 (35.7)	0 (0)	
	Resolved	12 (63.2%)	8 (57.1)	4 (80)	0.468
Native	No response	5 (20%)	4 (18.2)	1 (33.3)	
	Improved	9 (36%)	8 (36.4)	1 (33.3)	
	Resolved	11 (44%)	10 (45.5)	1 (33.3)	0.504

N (%), compared by Fisher exact test.

PUD, peptic ulcer disease.

## DISCUSSION

This is one of the first studies to compare the impact of HP infection and therapy in non-native children versus native pediatric patients. We note a relative risk of HP diagnosis in non-native patients 3 times that of native-born children and adolescents. This elevated risk in non-natives is particularly compelling, considering recent findings linking even asymptomatic HP carriage with an increased lifetime risk of gastric cancer (11). Accordingly, these data, although representing a single-center experience, clearly suggest a significant disparity in HP burden between native and non-native pediatric populations.

Our overall HP infection rate was 15.6% in patients who underwent EGD for GI symptoms, similar to previously reported HP prevalence of 3%–21% in the pediatric population of North America and Europe (9,10). However, when analyzed by nativity status, the rate increased to 39.1% in the non-native group, mirroring the substantially higher HP prevalence of 33%–79% in developing countries (12,13). We also observed that the endoscopic disease activity score was higher in the non-native group, suggesting the presence of more severe HP variants in this population. One possible explanation is the higher incidence of pathogenicity islands in the global south, where the bulk of our non-native patients were born (2,3,11,14,15). This severity and risk may also be impacted by genetic factors, as prior work has suggested that the risk of HP-associated gastric cancer varies by race inside the same geographic area (16).

Our findings also demonstrate higher endoscopic disease activity in non-native populations and confirm prior work demonstrating empiric therapy produces remarkably strong outcomes, as shown by 85% symptomatic resolution in all comers (17). Patients received 14 days of amoxicillin, clarithromycin, and omeprazole. Current guidelines recommend susceptibility-guided therapy in pediatric patients due to the belief that there is an unacceptable failure rate after empiric therapy (4). However, recent work in adults examining outcomes of empiric therapy per protocol suggests very high eradication rates using standard therapy with high-dose amoxicillin (18–20). However, there is a paucity of data with regard to the efficacy of empiric versus guided therapy in pediatric patients, (in particular examining high-dose amoxicillin-containing treatments) and there remains a significant risk of the development of resistance, which targeted therapy can mitigate (21). In aggregate, our results and the available data suggest that further study of this topic in pediatric patients is needed.

The primary arguments for the current North American Society for Pediatric Gastroenterology, Hepatology, and Nutrition guidelines recommendation of endoscopic confirmation of HP infection are the risk of a coincidental HP infection and the need for culture- and sensitivity-guided therapy. However, our findings suggest that an increase in risk in non-native pediatric cohorts, in the context of current poor guideline adherence and recent work increasing concern for negative outcomes such as gastric cancer in even asymptomatic carriage, warrants a prospective study of fecal antigen testing or breath testing alone as an adequate diagnostic measure (22). Stool HP antigen testing (an immunoassay capturing the presence of active HP colonization) remains one of the more common methods to assess for an HP infection, and it has been noted to have an extremely strong sensitivity and specificity in pediatric patients (96.6%–98% and 94.7%–100%, respectively (23,24)). As technology develops, in particular with the advent of next-gen sequencing approaches to determine antibiotic sensitivity (25), it suggests reassessment and further study of our approach in pediatrics are indicated.

There are several limitations of our study. First, this is a single-center retrospective study. This is partially beneficial, in that our population includes many non-native patients of a similar demographic makeup to native patients, but also given regional patterns of HP pathogenicity, this may limit generalizability to other non-native populations. It also has limited our sample size. We were unable to assess HP antibiotic sensitivity due to the relatively low number of cultures sent for evaluation, and we were not able to capture the bulk of repeat testing as many patients undergo repeat stool antigen tests at their Primary Care Provider, which is not recorded in our records. We were also unable to obtain clear records of parental or familiar HP infection or gastritis history. Our patients also displayed multiple additional risk factors for HP (low socioeconomic status and large percentage of African and Hispanic descent). This may lead to higher-than-expected positivity rates overall, but the well-matched nature of the demographics of our native/non-native cohort allows our comparison of the nativity as a risk factor to stand. A larger multicenter prospective study involving various regions is needed in the future, as well as a prospective study to answer questions raised regarding the need for endoscopic diagnosis and sensitivity-guided therapy in pediatric patients.
